# Strongyloidiasis—An Insight into Its Global Prevalence and Management

**DOI:** 10.1371/journal.pntd.0003018

**Published:** 2014-08-14

**Authors:** Santhosh Puthiyakunnon, Swapna Boddu, Yiji Li, Xiaohong Zhou, Chunmei Wang, Juan Li, Xiaoguang Chen

**Affiliations:** 1 Key Laboratory of Prevention and Control for Emerging Infectious Diseases of Guangdong Higher Institutes, Department of Pathogen Biology, School of Public Health and Tropical Medicine, Southern Medical University, Guangzhou, Guangdong, China; 2 Department of Traditional Chinese Medicine, Nanfang Hospital, Southern Medical University, Guangzhou, Guangdong, China; George Washington University, United States of America

## Abstract

**Background:**

*Strongyloides stercoralis*, an intestinal parasitic nematode, infects more than 100 million people worldwide. Strongyloides are unique in their ability to exist as a free-living and autoinfective cycle. Strongyloidiasis can occur without any symptoms or as a potentially fatal hyperinfection or disseminated infection. The most common risk factors for these complications are immunosuppression caused by corticosteroids and infection with human T-lymphotropic virus or human immunodeficiency virus. Even though the diagnosis of strongyloidiasis is improved by advanced instrumentation techniques in isolated and complicated cases of hyperinfection or dissemination, efficient guidelines for screening the population in epidemiological surveys are lacking.

**Methodology and Results:**

In this review, we have discussed various conventional methods for the diagnosis and management of this disease, with an emphasis on recently developed molecular and serological methods that could be implemented to establish guidelines for precise diagnosis of infection in patients and screening in epidemiological surveys. A comprehensive analysis of various cases reported worldwide from different endemic and nonendemic foci of the disease for the last 40 years was evaluated in an effort to delineate the global prevalence of this disease. We also updated the current knowledge of the various clinical spectrum of this parasitic disease, with an emphasis on newer molecular diagnostic methods, treatment, and management of cases in immunosuppressed patients.

**Conclusion:**

Strongyloidiasis is considered a neglected tropical disease and is probably an underdiagnosed parasitic disease due to its low parasitic load and uncertain clinical symptoms. Increased infectivity rates in many developed countries and nonendemic regions nearing those in the most prevalent endemic regions of this parasite and the increasing transmission potential to immigrants, travelers, and immunosuppressed populations are indications for initiating an integrated approach towards prompt diagnosis and control of this parasitic disease.

## Introduction

*Strongyloides stercoralis*, one of the most common and globally distributed human pathogens of clinical importance, infects 30–100 million people worldwide [1]. *S. fuelleborni*, another species of the same genus, is found sporadically in Africa and Papua New Guinea [2]. Strongyloidiasis is endemic in Southeast Asia, Latin America, sub-Saharan Africa, and parts of the Southeast United States [3]. Unique characteristics of this nematode are its immense ability to persist and replicate within a host for decades while producing minimal or no symptoms and its potential to cause life-threatening infections by dissemination and hyperinfection in debilitated and immune-compromised patients [4].

After its first report in 1876 from the feces of French soldiers with diarrhea who were returning from the old Indochina region, the disease was known for many years as “Cochin-China diarrhea” [5], which describes the most common gastrointestinal manifestations, such as epigastric pain and watery diarrhea, of this parasitic infection [6]. It took more than a century to trace most of the basic biology of this nematode and its extravagant ability to disseminate in host tissues, thereby leading to a spectrum of clinical complications.

In this review, we analyze the various case reports since 1970 from different parts of endemic and nonendemic foci of *S. stercoralis* to delineate a comprehensive global survey of this parasitic infection. We have focused more details on the different diagnostic methods followed by the investigators in various case reports and discussed some recent novel methods in serological and molecular diagnosis towards the aim of establishing guidelines for diagnosis to decipher the global prevalence of this disease.

## Epidemiology and Global Prevalence of Strongyloidiasis

Strongyloidiasis is an emerging global infection that is underestimated in many countries [7]. The prevalence of this disease has been on the increase, especially in southern, eastern and central Europe, the islands of the Caribbean, Southeast Asia, Latin America, and sub-Saharan Africa. In nonendemic regions of the world, it is mainly diagnosed in individuals who were prisoners during World War II and in immigrants from endemic countries [8]. Males, people working with soil (such as in coal mines and farms), people of white race, patients with altered cellular immunity (especially those on long-term steroid therapy), patients with lymphoma, allograft transplant recipients, travelers to areas of endemicity, and other institutionalized individuals are at the greatest risk of acquiring this disease [9], [10]. A strong association is seen between strongyloidiasis and concurrent immunosuppressive diseases such as human T cell lymphotropic virus-1 (HTLV-1) [11], human immunodeficiency virus (HIV) infection, and hematological malignancies [12], [13].

**Figure 1:**
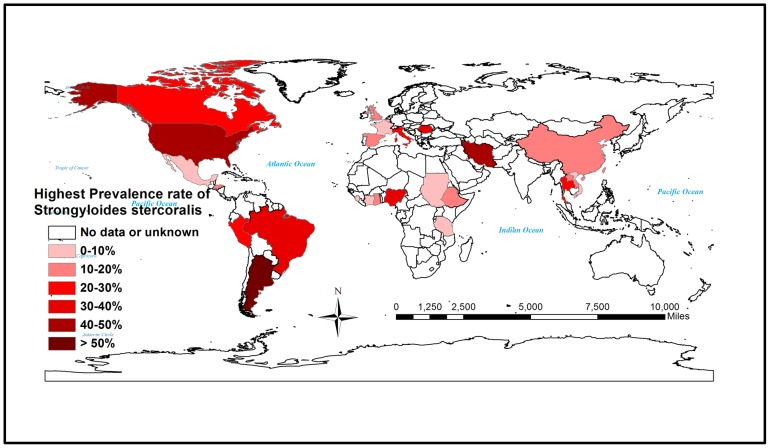
Map showing the global prevalence of *S. stercoralis* infection. The map was constructed using the data from the table, with the highest percentage prevalence of reported case studies and screening among populations from different countries that are endemic and nonendemic regions of the disease.

Global prevalence of *S. stercoralis* has been on the increase in the past few years, especially in many known endemic areas of the disease (Figure 1). The continued increase in infection rate is solely attributed to poor personal hygiene, insufficient drinking water supply, unsatisfactory sanitary measures, and lack of knowledge about the disease in high-risk populations. Many isolated case reports on the emergence of the disease in different parts of the world that are nonendemic for the disease are being published. Most of these case studies are associated with patients with immunosuppressive diseases, those on corticosteroid therapy, organ transplant recipients, and patients with hematological malignancies or other debilitating diseases. Newer diagnostics and endoscopies are being implemented widely to diagnose strongyloidiasis in many complicated clinical cases. Serological screening and molecular methods like polymerase chain reaction (PCR) are slowly becoming popular and are used in parallel with routine diagnostic screening methods. A comprehensive analysis of the case reports from different areas of endemicity and nonendemicity was carried out in an effort to highlight the importance of implementing the most appropriate diagnostic methods to delineate the global prevalence of this disease (Table 1).

**Table 1:** Global survey of prevalence of *S. stercoralis* in endemic and nonendemic regions of the disease.

Country/Location	Number of Specimens Analyzed	*S. stercoralis* Prevalence %	Year (References)	Diagnostic Methods Used in Reported Studies
Argentina	36	83.3	1993 [90]	Direct stool examination
Argentina	154	50.5	2003 [91]	Direct stool examination
Argentina	42	50	2010 [92]	Direct fresh stool examination, Ritchie's method, and agar plate culture (APC)
Argentina	225	29.4	2010 [70]	Agar plate culture, Harada-Mori filter paper culture, Baermann concentration and ELISA based on crude antigen (CrAg-ELISA), and LIPS
Brazil	634	6.6	2011 [93]	Agar plate culture, Baermann-Moraes (BM), and spontaneous sedimentation
Brazil	160	1.3	2008 [94]	Baermann methods modified by Moraes and Lutz
Brazil	503	6.7	2008 [95]	Direct stool examination using the techniques of Faust, Lutz, Rugai, et al.
Brazil	120	30.1	2006 [96]	Baermann and Hoffman methods, indirect fluorescent antibody (IFA) test, ELISA, and western blotting
Brazil	200	2.5	1999 [97]	Direct stool examination
Brazil	900	13	1998 [98]	Baermann-Moraes and Lutz methods
Brazil	650	3.85	1999 [13]	Direct stool examination
Brazil	343	15.45	1995 [99]	Direct stool examination
Belgium	2,591	0.92	2009 [74]	Direct stool examination, Baermann concentration method, antigen detection and multiplex real-time PCR
Cote d'Ivoire	6,952	0.1	2006 [100]	Kato-Katz (K-K) technique
Canada	232	24.7	1990 [101]	Direct stool examination and ELISA
Canada	1,605	0.43	1993 [102]	Stool examination and serological tests
China	1,397	14	2012 [14]	Direct stool examination and other clinical specimens from patients
Ethiopia	1,239	13	2000 [103]	Direct stool examination and Baermann methods
Ethiopia	384	12	2010 [104]	Direct saline mount, formol-ether and water-emergence techniques
Ethiopia	378	7.4	2009 [105]	Direct stool examination, formol-ether technique
France	1,001	1.4	1997 [106]	Direct analyses by Kato and Ritchie methods
France	800	6.4	1996 [107]	Direct analysis by Kato-Katz method and routine microscopy
Ghana	212	17.9	2009 [73]	Baermann sedimentation tests, duplicate coprocultures, and real-time PCR
Honduras	427	16.4	1993 [108]	Direct smear, a modified Baermann technique, and agar plate culture
Italy	5,351	0.07	2011 [109]	Direct microscopy, culture, and ELISA
Italy	132	28	2009 [110]	Indirect fluorescent antibody test
Iran	13,915	0.03	2009 [111]	Modified formalin-ethyl acetate sedimentation technique accompanied with trichrome stain
Iran	782	2.04	2011 [71]	Agar plate culture and PCR (single and nested)
Iran	150	42	2010 [112]	Stool examination by formalin-ether and Kato-Katz techniques
Israel	106	0.9	1992 [113]	Direct stool examination and ELISA
Jamaica	312	24.2	1995 [86]	Stool examination and serology
Kuwait	11,230	0.46	2004 [114]	Direct stool examination and ELISA
Laos PDR	664	19	1998 [115]	Agar plate culture and Kato-Katz thick smear method
Mexico	200	1	1997 [116]	Direct stool examination
Nigeria	4,470	35.2	1997 [117]	Direct stool examination
Nigeria	227	5.3	2004 [118]	Stool wet preparation and formol-ether concentration methods
Peru	83	28.91	1999 [119]	Baermann concentration technique modified by Lumbreras
Peru	256	0.87	2009 [120]	Direct microscopy of feces and by rapid sedimentation technique
Romania	294	6.9	1995 [121]	Direct stool examination
Romania	35	30	1989 [122]	Direct microscopy, culture, and Jejunal biopsy
Spain	250	12.4	2003 [123]	Detection of larvae of triple stool samples
Spain	16,607	0.9	2001 [124]	Direct microscopy, agar plate culture, and tissue biopsy
Sierra Leone	1,164	3.8	1995 [125]	Direct stool examination
Sudan	275	3.3	1998 [126]	Formol-ether concentration techniques
Thailand	491	11.2	1989 [127]	Direct stool examination
Thailand	332	28.9	2003 [128]	Agar plate culture technique and modified formalin-ethyl acetate concentration technique (MFECT)
Thailand	100	28	2010 [49]	Formalin-ether concentration technique (FECT)
Thailand	1,085	22.2	1999 [129]	Direct smear, agar plate culture, formalin-ether sedimentation technique, and the filter-paper method
Tanzania	368	10.2	2009 [130]	Kato-Katz, Koga agar plate, and Baermann techniques
Tanzania	342	10.5	2008 [131]	Kato-Katz, Koga agar plate, and Baermann techniques
Tanzania	1,078	1.6	2009 [132]	Direct stool examination and formalin-ether concentration methods
United Kingdom -Liverpool	2,072	12	2004 [19]	Microscopy and culture of stool or duodenal fluid and ELISA
US–North Carolina	172	7.5	2009 [133]	Direct stool microscopy and serology
US–Tennessee	225	8.4	1992 [134]	Agar plate and formalin-ethyl acetate concentration method
US–Kentucky	125	4	1965 [135]	Stool examination
US–Kentucky	561	3	1982 [10]	Stool examination
US–Kentucky	3,271	2.5	1982 [10]	Stool examination
US–Tennessee	575	4	1987 [32]	Stool examination
US–West Virginia	4,566	0.4	2000 [136]	Concentration techniques followed by iodine-stained smear examination and sputum culture
US–Maryland	51	3.9	1981 [137]	Stool examination
US–Maryland	339	0.6	1986 [138]	Single direct stool examination
US–Louisiana	8,458	0.4	1974 [139]	Stool examination by direct and concentration methods
US–Chicago, Illinois	358	1.7	1975 [140]	Single direct stool examination
US–New York	10,072	1	1975 [141]	Stool concentration method and sputum examination for larvae
US–Seattle, Washington	201	2.5	1995 [142]	Direct stool examination
US–Atlanta, Georgia	150	46	2007 [143]	Direct stool examination and serology
US–Ohio	700	3.71	1987 [144]	ELISA and fresh stool examination for larvae
US–Maryland	128	38	1987 [145]	Stool examination and Serology
US–Minneapolis, Minnesota	1,291	11.69	2007 [39]	Direct stool examination
Vietnam	3,197	0.84	1970 [146]	Direct stool examination and sedimentation technique

A statistical analysis carried out in our lab [14] showed a total of 106 detailed cases reported from China since the first documented case from Guangxi Province in 1973 up until 2012. A total of 67 cases were reported in the past 10 years (2001 to 2011), which exceeds the cumulative cases reported in the 30 years before this period and indicates the increasing rate of emergence of strongyloidiasis in China.

Globally, prevalence rates of strongyloidiasis are as high as 50% in certain areas where moist soil and improper disposal of human waste coexist, especially in West Africa, the Caribbean, Southeast Asia, tropical regions of Brazil, Cambodia, and temperate regions of Spain [15]. Southeast Asia appears to have the highest endemic percentage, and it is highly prevalent in some tropical aboriginal communities in Australia [16]. Although strongyloidiasis is uncommon in the United States, endemic foci exist in rural areas of the southeastern states and the Appalachian region (especially in eastern Tennessee, Kentucky, and west Virginia) and in Puerto Rico [17]. A higher prevalence rate is seen among patients in long-term institutionalized care (mental health facilities and prisons), in immigrants and refugees from tropical and subtropical countries [18], and in veterans of World War II and the Vietnam War [19].

Among the immigrant population, a high prevalence rate of 38% was reported in Southeast Asian immigrants in Washington, D.C. [20]. A Canadian epidemiological study revealed 11.8% incidence of infection in the Vietnamese population and a much higher seroprevalence of 76.6% in Cambodian immigrants [21]. Sudanese Lost Boys and Girls and Somali Bantu refugees demonstrated 46% and 23% seropositive rates, respectively [22]. High rates of larva currens are reported in Latin America. A stool serosurvey conducted in a community in the Peruvian Amazon region found an 8.7% incidence rate of *S. stercoralis* [23]. Although strongyloides infection is represented in all ages, infection initially occurs in childhood, as children are more likely to play outdoors in contaminated soil with bare feet [24].

Most of the epidemiological studies to assess the prevalence of infection in a population were carried out by microscopic screening of the stool samples (either single or multiple) to find larvae or by one of the concentration and culture methods. The discovery of positive cases has been increased in many studies after implementing serological screening or by using molecular methods in immunocompromised patients or high-risk groups. Recently, a real-time PCR method targeting the small subunit of a ribosomal RNA (rRNA) gene was developed for the detection of strongyloides DNA in fecal samples, including an internal control to detect inhibition of the amplification process. Novel methods of diagnosis like luciferase immunoprecipitation system assays based on recombinant antigens that are 100% sensitive may be a promising alternative to routine diagnostic methods which are less sensitive. These newer methods may hopefully enhance routine diagnosis of *S. stercoralis* infection in the future.

## Biology of the Parasite

Genus *Strongyloides* is classified in the order Rhabditida, and most of the 52 species are soil-dwelling, microbiverous nematodes that do not infect human beings. Other than *S. stercoralis* and *S. fuelleborni*, two more species, *S. myopotami* and *S. procyonis*, are reported to infect animal hosts and may be responsible for zoonotic infections [25]. The adult female worm is a slender, almost transparent worm that measures 2.2 to 2.5 mm long, has a diameter of 50 µm, and lives in tunnels between enterocyte in the small intestine. Parasitic males, although they do exist, do not have any role in human infections and are easily eliminated from the intestine [26], [27].

## Pathophysiology and Life Cycle

**Figure 2:**
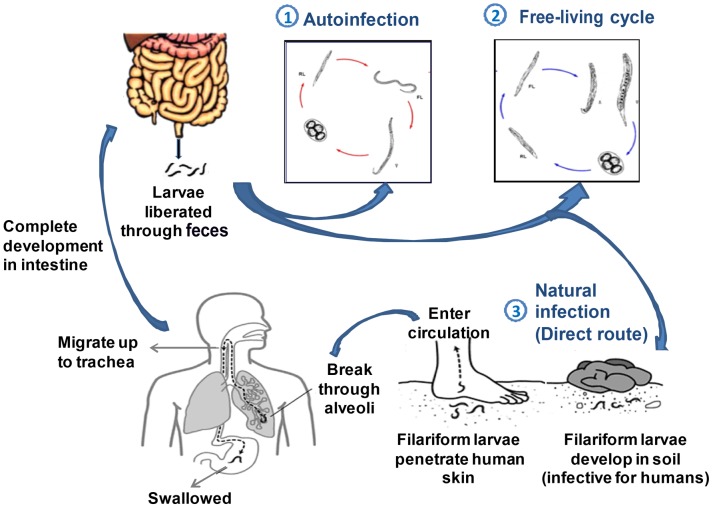
Life cycle of *S. stercoralis* with the heterogonic and parasitic phases.

*S. stercoralis* exhibits a complex and unique developmental phase with two distinct life cycles: a free-living heterogonic cycle and a parasitic life cycle completed in the same host [28]. In the free-living phase, during the development of nonparasitic adults, both males and females occur in soil, which maintains infestation of the soil (Figure 2). The parasitic phase allows noninfective new larvae to molt in the human host into infective filariform larvae, which then penetrate the intestine and set up a new cycle, leading to autoinfection or hyperinfection to increase the worm burden without exogenous reinfection [29]. This autoinfective phase is responsible for the decade-long persistence of infection in untreated hosts.

Human infection is mainly acquired by the filariform larvae penetrating the skin or mucous membranes either from autoinfection or from infected soil by a fecal-oral route [30]. At their portal of entry, larvae usually cause petechial hemorrhagic rash, which is followed by intense pruritis, edema, and congestion [31]. Larvae migrating through lymphatics and venules reach pulmonary circulation and produce hemorrhages in pulmonary capillaries. The larvae make their way farther into alveolar spaces and cause inflammatory responses associated with eosinophilic infiltration terminating in pneumonitis [32]. Finally, larvae crawl up the respiratory tract and are swallowed, thereby reaching the intestine.

Maturation of larvae into adult females occurs in the small bowels after two molts, and the emerged females produce eggs via parthenogenesis [33]. These parasitic females may live up to five years to continue their reproductive cycle. The eggs hatch in the intestine into noninfective rhabditiform larvae, which may be passed through stool into the environment to continue the heterogonic free-living phase in the soil. The excreted larvae in stool thus form the mainstay of laboratory diagnosis of infection and are also responsible for autoinfection after transforming into infective filariform larvae [34].

### 1. Autoinfection

Autoinfection is unique to *S. stercoralis* within the genus and also among other genera of intestinal parasites of vertebrates and accounts for it being a serious pathogen of humans. Premature transformation of noninfective larvae into infective larvae establishes a parasitic developmental phase within the host, and it can be maintained throughout the host's life by repeated migratory cycles without exit from the primary host [30]. Infective filariform larvae reenter circulation by penetrating the mucosa of the colon or small intestine and cause internal autoinfection or the larvae penetrate the perianal skin and cause external autoinfection [28]. External autoinfection in most cases leads to the development of larva currens. After entering the circulation, larvae are carried to the lungs and repeat the cycle, which accounts for the frequent recurrence and chronicity of disease in migrants to endemic areas of disease [35]. Autoinfection, which is otherwise kept in check by the host immune response in healthy individuals, usually occurs in patients with impaired cell-mediated immunity [36]. Autoinfection gives rise to the two most severe forms of strongyloidiasis: hyperinfection syndrome (HIS) and disseminated strongyloidiasis (DS).

### 2. Hyperinfection syndrome

Hyperinfection syndrome denotes a phenomenon in which a tremendous increase in the number of worms leads to excessive worm burden without the spread of larvae outside the usual migration pattern. The worms are detectable in extraintestinal regions, especially in the lungs, and the detection of larvae in stool and/or sputum is the hallmark in diagnosis of hyperinfection [37]. Generally, the hyperinfection ensues from the enormous multiplication of infective larvae and their migration in the immunosuppressed state, but it is not always true, as some authors have also described hyperinfection syndrome in immunocompetent patients [38].

The clinical manifestation of hyperinfection syndrome is classified, based on the origin, into gastrointestinal and extraintestinal disease mainly involving the respiratory tract. Risk factors for developing hyperinfection include corticosteroid therapy, stem-cell transplantation, alcoholism, HIV, and HTLV-1 infection. Pulmonary symptoms such as pulmonary infiltrates, diffuse alveolar hemorrhage (DAH), and respiratory failure develop in patients, which, if not treated, turn out to be fatal. The high mortality in hyperinfection is often due to negligence and lack of familiarity of health care providers in recognizing the need for parasite screening before advocating empirical corticosteroid therapy [39].

### 3. Disseminated strongyloidiasis

Disseminated strongyloidiasis involves widespread dissemination of larvae to extraintestinal organs that are outside the realm of the parasite's ordinary life cycle. Multiple organs are affected, including the lungs, liver, heart, kidneys, endocrine organs, and central nervous system [40]. In severe disseminated disease, translocation of enteric bacteria may lead to polymicrobial bacteremia and occasionally meningitis with enteric pathogens. The bacteria may be carried on the migrating filariform larvae or may enter the circulation through intestinal ulcers. Major complaints are fever, abdominal pain and distension, weight loss, vomiting, cough, anemia, and hemoptysis [41]. Disseminated strongyloidiasis may not always occur as a fatal outcome of hyperinfection syndrome. Disseminated infection with bloody pericardial effusion has been reported in a nonimmunosuppressed patient without manifestations of hyperinfection syndrome [42]. This clinical finding is relatively common in high-risk populations, which are frequently misdiagnosed with gram-negative septicemia or acute respiratory distress syndrome.

### 4. Acute and chronic strongyloidiasis

In acute strongyloidiasis, patients become symptomatic immediately after exposure, and the symptoms may last up to several weeks [43]. Acute infection is generally characterized by gastrointestinal (GI) and pulmonary symptoms. GI symptoms such as diarrhea, anorexia, and abdominal pain begin about 2 weeks after infection, with larvae detectable in stool after 3 to 4 weeks. Pulmonary symptoms such as tracheal irritation, cough, and bronchitis begin much earlier than GI symptoms as larvae migrate through the lungs within a few days after exposure. Chronic infection with *S. stercoralis* is often asymptomatic [44]. In symptomatic patients, major complications are due to chronic gastrointestinal manifestations such as diarrhea, constipation, and intermittent vomiting. Dermatological manifestations like urticarial, petechial, and purpuric rashes and larva currens are also common.

## Diagnostic Challenges

The diagnosis of strongyloidiasis requires a high degree of suspicion, as most of the patients with the infection do not show distinctive clinical features and laboratory and imaging findings often turn out to be nonspecific [45]. Multiple clinical findings appear with fatal outcomes after treatment with steroids for a severe disease of unknown etiology and are later confirmed as a case of disseminated strongyloidiasis. Clinicians should be aware that the clinical spectrum of infection may lead to pulmonary infiltrates, acute respiratory distress syndrome, small bowel obstruction, and multisystem organ failure. Clinical correlation of the symptoms with travel and residence history and imported strongyloidiasis should be considered in travelers to and immigrants from endemic areas. Furthermore, the possibility of strongyloidiasis should always be considered in any immunocompromised patients who suddenly deteriorate; delay in diagnosis frequently results in death, despite intense treatment [16].

Diagnosis of strongyloides hyperinfection is relatively easy because of the high number of larvae that are seen in stool smears and usually seen in sputum. Many have reported unexpected findings of the larvae in ascites fluids and blood smears [46]. Direct stool examination in saline and Lugol's iodine stain has been used for mass screening of a population in many epidemiological surveys, but a single direct stool examination alone is always inadequate, as evidenced by many reports of hyperinfection with negative stool screening exams. As the egg output compared to other parasitic helminths is too low, a single stool exam is only about 50% sensitive for making diagnosis and even lower in chronic asymptomatic patients [47].

Other methods of diagnosis include Baermann's and formalin-ethyl acetate concentration techniques, with improved sensitivity of stool exams. Blood agar plate culture method is also preferred due to high sensitivity and ease of implementation. In a comparative study carried out to determine the efficacy of diagnostic methods, agar plate culture method (APC) has shown high sensitivity, more than 96% compared to direct fecal smear, formalin-ether concentration techniques, and Harada Mori's filter paper culture methods [48]. It is essential to examine stool samples repeatedly to achieve correct diagnosis, and a negative result does not always indicate the absence of infection [47], [48]. Peripheral eosinophilia (>600/mL) represents an immune response to larvae migrating through host tissues and is common during acute infection (as high as 75% to 80%), intermittent during chronic infection (often the only abnormal laboratory test), and frequently absent in severe strongyloidiasis and in the immunocompromised host [44].

### 1. Microscopy and culture of stool

*S. stercoralis* larvae are secreted in feces, and the finding of them may be intermittent. Larvae are usually passed in stool approximately 1 month after skin penetration. The eggs of the parasite are not usually found in feces; instead, they hatch immediately to larvae within the intestine, and the larvae are released in stool to undergo the heterogonic phase of development in soil [49]. *S. fuelleborni* infection in children sheds eggs rather than larvae in feces and is easily diagnosed using microscopic techniques. Direct smear examination of stool in saline and Lugol's iodine stain has been used to discern larvae in stool and is performed as a definitive diagnostic test. Most epidemiological screenings rely only on this method even though direct wet mounts yield very low larvae (sensitivity up to 30%) on many occasions. Hence, it is mandatory to screen multiple samples, and this should be performed with one of the concentration methods, which may increase sensitivity up to 70% to 80%. More than 90% sensitivity for stool samples is seen if seven or more samples are examined [50].

Concentration methods like formalin-ethyl acetate, Harada-Mori techniques, and Baermann concentration increase the yield and are much more sensitive than single stool smears [51]. A modified formalin-ether concentration technique using fresh stool without a preservative, a short-term formalin exposure, use of wire mesh instead of gauze, and a five-minute centrifugation has shown more efficiency in larval yield compared to the conventional method with the usual parameters [49]. In the Harada-Mori technique, filter paper containing fresh fecal material is placed in a test tube with water that soaks the filter paper by capillary action. Incubation at 30°C provides conditions suitable for the development of larvae, which migrate to the filter paper [47].

Baermann concentration method exploits the tendency of worms and larvae to migrate from a solid into a surrounding liquid medium (hydrotropism) when stimulated by a slightly elevated temperature (thermotropism) and then to settle to the substratum. In this technique, a comparatively large amount of sample can be screened, and the chances of finding different larval stages as well as adults are higher [52]. In the agar culture method, the stool sample is placed on the nutrient agar or blood agar plate and incubated for 48 hours. Serpiginious tracks of bacterial growth along the paths of crawling larvae become apparent after 1 to 2 days of incubation, and motile larvae can be easily visualized with the aid of a dissecting microscope [47], [53]. Agar plate culture method is preferred due to its high sensitivity and ease of implementation in standard microbiological laboratories and also for detection of larvae from samples like sputum, bronchial aspirates, and other body fluids [54]. Although laborious and time consuming, it is proven to be more than four times as efficient as direct smear procedures for detection of larvae in stool [48].

String test (Entero test), once popular, is seldom used for collection of larvae from the patient's duodenum [55]. The reported sensitivity ranges from 40% to 90%. This method, which creates inconvenience for the patient, is gradually being replaced by more sensitive duodenal aspiration or histological examination of duodenal or jejunal biopsy [54], [56].

### 2. Endoscopy and histological features

Gastrointestinal endoscopy may range from normal-appearing mucosa to severe duodenitis and colitis showing edematous mucosa, white villi, and erythematous mucosa. In most cases of pulmonary hyperinfection, the larvae are identified with duodenal biopsy. Duodenal biopsy and histopathologic examination identified larvae in 71.4% of immunosuppressed patients [57]. Thus, in addition to stool analysis, endoscopic observation and biopsies are very important [58]. In disseminated strongyloides infection, larvae can be recovered from extraintestinal sites, including sputum, bronchoalveolar fluid [59], cerebrospinal fluid (CSF) [60], urine [61], ascites, gastroesophageal biopsy, and skin biopsy [62], [63]. CSF analysis shows elevated protein levels, decreased glucose levels, and pleocytosis with neutrophilic predominance, and a gram stain performed may exhibit various bacterial florae [62], [64]. A wet mount preparation of the CSF will usually reveal *S. stercoralis* larvae.

### 3. Serological testing

Serological assays are now widely available; moreover, due to increased sensitivity, they have the potential to be used in multiple helminthic infections. Several immunodiagnostic methods have been developed over the years with limited success, including skin testing with larval extracts, indirect immunofluorescence using fixed larvae, filarial complement fixation testing, radioallergosorbent testing for specific immunoglobulin E (IgE), gelatin particle indirect agglutination (GPIA), western blot analysis, and enzyme-linked immunosorbent assay (ELISA) for immunoglobulin G (IgG) antibodies [47]. Strongyloides-specific antibodies may be used for serologic follow-up, which may indicate seroconversion after successful therapy. ELISA testing has been shown to detect the disease in approximately 85% to 90% of patients (sensitivity of 82% to 95%) [65]; however, its sensitivity may be lower in severely immunocompromised patients and does not distinguish past and present infection in endemic areas of disease.

The strongyloides antibody test shows cross-reactivity with other helminth infections such as filariasis, ascariasis, and acute schistosomiasis. These antibodies can persist for a long time in the host; hence, a differential diagnosis of symptomatic strongyloidiasis is unavoidable in most endemic areas of this disease [66]. An enzyme immune assay for anti-strongyloides IgG may be a good option for rapid diagnosis when a stool examination result is negative as well as in immunosuppressed patients.

A luciferase immunoprecipitation assay described recently may eventually prove to be more accurate than ELISA testing [67]. This luciferase immunoprecipitation system (LIPS) was developed against an antibody to a recombinant strongyloides antigen (NIE). When compared with NIE-ELISA, LIPS showed improved specificity and after a second antigen, *S. stercoralis* immunoreactive antigen (SsIR), was used in combination, it resulted in a 7-fold difference between positive and negative values. Moreover, it did not show any signs of cross-reactivity with serum from filarial infected patients, which accounts for a major drawback in most serological assays. If made cost-effective and widely available, LIPS could be a substitute used in the effective screening of patients, performing more rapidly and specifically than ELISA. In a comparative evaluation study conducted in endemic regions of Thailand, the gelatin particle agglutination test also was judged to be more practical as a screening test than conventional ELISA [68].

### 4. Molecular diagnosis

Polymerase chain reaction assays for intestinal parasites, including strongyloides, are increasingly being used on fecal DNA samples, with enhanced specificity and sensitivity of detection. In a recently conducted study, multiplex PCR reactions with specific primers for protozoa and another primer set for helminthes were used. The PCR products obtained were hybridized to beads linked to internal oligonucleotide probes and detected on a Luminex platform. This multiplex PCR bead assay showed 83% and 100% sensitivity and specificity compared with parent multiplex real-time PCR assays and provides a sensitive diagnostic screen for a large panel of intestinal parasites [69].

Most conventional serological diagnosis, like ELISA, is based on crude antigen from parasite extracts. Newer techniques such as the luciferase immunoprecipitation system assays based on recombinant antigens were devised and showed the highest specificity (97.8%) and 100% sensitivity [70] when compared with conventional ELISA. Molecular diagnosis based on detection of specific copro-DNA in stool by a single PCR method amplifying a short (100 base pair) target showed greater efficiency (100%) for detection of *S. stercoralis* in fecal samples compared to agar plate culture and nested PCR, which amplifies a larger target [71]. In most PCR-based assays, a frequently observed factor that decreases the efficiency of detection is the presence of PCR inhibitors, which are relatively common in stool samples; this is more critical for assays of samples with lower parasite DNA [72]. This is resolved by increasing the amount of feces used for the PCR assay by concentration of stool by acid-ether prior to DNA extraction [71].

A real-time PCR method targeting the small subunit of the rRNA gene was developed for detection of *S. stercoralis* DNA in fecal samples. The use of this assay could facilitate monitoring the prevalence and intensity of infection in helminth interventional programs, as this real-time PCR also includes an internal control to detect inhibition of the amplification process by fecal contaminants [73]. This assay showed a 2-fold increase in the detection rate when compared with the Baermann sedimentation method and could be an alternative to less sensitive conventional screening methods.

In another recent study, a multiplex real-time PCR, when compared with microscopic examination and antigen detection in travelers and migrants, showed increased detection rates from 0.1% to 0.8% [74]. A growing number of routine diagnostic laboratories are implementing multiplex real-time PCR for detection of intestinal parasites, and these assays can be extended specifically to screening travelers and migrants for *S. stercoralis*. If implemented effectively, these emerging new technologies certainly may enhance the diagnosis of strongyloides infection in the future.

## Prevention and Treatment Strategies

The aim of pharmacotherapy in strongyloidiasis is to eradicate the infection, reduce morbidity and mortality, and prevent hyperinfection and disseminated complications. Several antihelminthic drugs are available for this purpose, though few are recommended for established infection. Most of these drugs allow selective interference in the biological and metabolic pathways of the adults in relatively small doses. The effectiveness of many of these antihelminthic agents against larvae is poor, and they are more effective only when established infection occurs.

Thiabendazole, albendazole, and mebendazole are used for the treatment of acute and chronic strongyloidiasis [75] but showed varied results in many drug trials [76]. Albendazole has a high-affinity binding capacity to free beta-tubulin in parasite cells, thereby inhibiting tubulin polymerization. This eventually results in loss of cytoplasmic microtubules and thus decreases adenosine triphosphate (ATP) production in worms, ultimately leading to energy depletion, immobilization, and death. Mebendazole inhibits microtubule formation and causes worm glucose depletion but shows variable efficacy against strongyloides. Even though used successfully to treat a number of patients with *S. stercoralis* hyperinfection, decreased accessibility due to poor absorption of the drug by migrating larvae may be the reason for its variable efficacy [77]. These benzimidazoles not only kill adult gut-dwelling stages of the parasite but also sterilize the larvae and eggs to some extent. Thiabendazole was a therapeutic option for strongyloidiasis for quite a long time but has been discontinued due to its unfavorable side effects [75], [78].

Ivermectin has now emerged as the drug of choice for acute and chronic strongyloidiasis in intestinal stages, hyperinfection syndrome, and disseminated strongyloidiasis [79]. It is a semisynthetic derivative of the macrolide mold product avermectin, which binds selectively to glutamate-gated chloride ion channels of invertebrate nerve and muscle cells, thereby increasing the cell membrane permeability with hyperpolarization and causing paralysis and cell death. In patients who are too sick to tolerate or absorb oral (PO) ivermectin, rectal (PR) or subcutaneous (SC) dosing may be effective [80], [81]. The eradication rates with ivermectin in many drug trials showed remarkable results and up to a 97% cure rate with even a 2-day course in asymptomatic cases, but in patients with hyperinfection and dissemination, daily drug administration until symptoms resolve with negative laboratory tests for larvae for at least two weeks is recommended [82]. The World Health Organization (WHO)-recommended treatment for strongyloidiasis is either ivermectin (200 µg/kg bodyweight in a single dose) or albendazole (400 mg daily for 3 days) [83]. In a study carried out in Zanzibar, the effect of the two regimens was compared: a cure rate of 82.9% was achieved with ivermectin, while three doses of albendazole cured only 45% of infected individuals [79].

Combination therapy including ivermectin and thiabendazole has been shown to be superior to albendazole alone, and ivermectin is becoming the drug of choice because it has fewer unfavorable side effects [79], [84] and a better rate of larval clearance from stool [75]. A newer drug, tribendimidine, remains under investigation in China and shows some promise in the treatment of strongyloidiasis [85].

Patients with hyperinfection are highly infectious and should be treated in isolation because sputum, stool, vomitus, and other body excreta may contain infective (filariform) larvae. Screening family members [86] and the use of universal precautionary measures to prevent spread of infection should be followed by all close associates of the patient, including health care workers [87]. Antibiotic therapy directed towards enteric pathogens should be provided if bacteremia or meningitis is present. Steroids and the use of leukotriene synthesis inhibitors should be avoided; they will worsen the infection because leukotrienes play a potential role in immunity against strongyloides infection [88]. Empiric corticosteroid administration may cause life-threatening hyperinfections, usually in immunosuppressed patients; hence, it should be avoided to the extent that it is possible [39]. Surgical interventions may be required in rare instances of acute abdominal symptoms (peritonitis) due to bowel obstruction or infarction in severe strongyloidiasis. Patients with hyperinfection syndrome often have complications of sepsis, shock, and acute respiratory distress syndrome (ARDS) and hence should receive care in a facility properly equipped for intensive management [89].

## Discussion

A definitive diagnosis of strongyloidiasis is usually made by the detection of larvae in stool. However, diagnosis is difficult because of low parasite load and irregular larvae output in the majority of subclinical infections; thus, the true prevalence is often underestimated. Though endemic in some developing countries, strongyloidiasis still poses a threat to the developed world. Increased infectivity rates almost nearing endemic rates of infection in many developed countries among immigrants and travelers and in veterans of war made the spread of infection imminent. In addition to natural methods of transmission in rural populations caused by poor personal hygiene and contemptible sanitary measures, the zoonotic transmission capacity makes the situation more serious, as domesticated small ruminants act as reservoir hosts of *S. stercoralis*. Novel diagnostic methods and treatment strategies with newer effective drugs are expected to improve epidemiological studies and control efforts for the prevention and treatment of strongyloidiasis. As most cases of hyperinfection syndrome and disseminated strongyloidiasis happen in immunocompromised individuals, especially those taking systemic steroids, clinicians should be aware of the risk factors associated with strongyloides infection prior to administering corticosteroid therapy. Awareness of increased predisposition to strongyloides infection is indispensable when gastrointestinal or pulmonary symptoms are observed in immunocompromised patients.

Many efficient integrated approaches are essential and, depending on the source of infection, infectivity rate in the population, and transmission capacity, should be implemented in the diagnosis, treatment, and management of this disease. This could be possible through a comprehensive analysis with an aim of understanding the infection in endemic regions of the disease and also by a thorough analysis of this emerging infection in immunocompromised populations and other risk groups in nonendemic regions of the disease.

**Top Five Papers in the Field**

1. Olsen A, van Lieshout L, Marti H, Polderman T, Polman K, et al. (2009) Strongyloidiasis—The Most Neglected of the Neglected Tropical Diseases? Trans R Soc Trop Med Hyg 103: 967–972.
2. Keiser PB, Nutman TB (2004) *Strongyloides stercoralis* in the Immunocompromised Population. Clin Microbiol Rev 17: 208–217.
3. Siddiqui AA, Berk SL (2001) Diagnosis of *Strongyloides stercoralis* Infection. Clin Infect Dis 33: 1040–1047.
4. Montes M, Sawhney C, Barros N (2010) *Strongylodies stercoralis*: There but Not Seen. Curr Opin Infect Dis 23: 500–504.
5. Knopp S, Mgeni AF, Khamis IS, Steinmann P, Stothard JR, et al. (2008) Diagnosis of Soil-Transmitted Helminths in the Era of Preventive Chemotherapy: Effect of Multiple Stool Sampling and Use of Different Diagnostic Techniques. PLoS Negl Trop Dis 2: e331.

**Key Learning Points in Risk Factors and Management of Strongyloidiasis**

- Patients with hyperinfection syndrome are highly infectious, as their secretions and body excreta may contain many infective filariform larvae. In rural areas, the disease has been reported, showing gathering in the family and a relation to living and working environment and poor personal hygiene. Occupationally related risks are more common in farmers, gardeners, coal mine workers, and health care workers.
- Patients with debilitating diseases such as diabetes, nephrotic syndrome, systemic lupus erythematosis (SLE), chronic obstructive pulmonary diseases, and carcinoma and recipients of organ transplantations have a high risk of developing fatal clinical forms of strongyloidiasis. Immunosuppressive patients with HTLV-1, HIV, or many hematological malignancies have shown concurrent strongyloides infection. Travelers and migrants to endemic areas and prisoners of wars in nonendemic areas are at greatest risk of acquiring the disease.
- Screening family members of patients, treatment in isolation, and following universal precautionary measures to prevent spread of infection to all close associates and to health care workers are mandatory. Improving the living conditions of rural population in areas of endemicity, providing a safe drinking water supply and good sanitary measures, and inculcating knowledge about the disease in high-risk populations help to reduce the infectivity rate in rural populations.
- Patients with bacteremia or meningitis following disseminated strongyloidiasis and hyperinfection should be treated with effective antibiotic therapy, and those developing complications of sepsis, shock, and acute respiratory distress syndrome should receive intensive care and critical support. Surgical interventions are suggested in acute cases with peritonitis, bowel obstruction, or infarction in severe disseminated strongyloidiasis.
- Empiric corticosteroid therapy in immunosuppressed patients often leads to life-threatening hyperinfection and severe strongyloidiasis. Prompt diagnosis of any strongyloides infection in immunocompromised patients is highly recommended prior to empiric corticosteroid therapy, which otherwise can lead to fatal complications and increased chances of mortality.

## Supporting Information

File S1 **Search strategy and selection criteria.**
(DOCX)

File S2 **Endemicity of Strongyloides infections in China.**
(DOCX)
