# Supplementary material for: Strongyloidiasis—An Insight into Its Global Prevalence and Management
Source: PLoS Negl Trop Dis. 2014 Aug 14;8(8):e3018. doi: 10.1371/journal.pntd.0003018 (PMC4133206; doi:10.1371/journal.pntd.0003018)
Supplement: File S1 — Search strategy and selection criteria. (DOCX) [file pntd.0003018.s001.docx]

**File S1.**

**Search strategy and selection criteria**

Data for this review were identified by a search in PubMed, CNKI (China National Knowledge Infrastructure) database, Science Direct and Springerlink without date restriction and using the key words “*Strongyloides stercoralis*” and “Strongyloidiasis”. We also reviewed many chapters of books from authors and WHO publications giving information about this helminth parasite. Many papers illustrating a specific point when encountered, we selected the most representative paper. Some of the information about the prevalence of strongyloidiasis from few endemic areas was published in other native languages than English, the abstract in English and translation of important information helped us to review those articles published in other languages.
