# Supplementary material for: Strongyloidiasis—An Insight into Its Global Prevalence and Management
Source: PLoS Negl Trop Dis. 2014 Aug 14;8(8):e3018. doi: 10.1371/journal.pntd.0003018 (PMC4133206; doi:10.1371/journal.pntd.0003018)
Supplement: File S2 — Endemicity of Strongyloides infections in China. (DOCX) [file pntd.0003018.s002.docx]

**File S2.**

**Endemicity of Strongyloides infections in China**

In China, according to National Human Parasite Distribution Survey (1996), Strongyloidiasis was prevalent in 26 provinces, mainly in the south with infection rate ranging from 0.12 to 14%. A statistical analysis of strongyloidiasis cases reported in China till 2012, since its first documented case in 1973 revealed a higher prevalence in our province, Guangdong (GD) which is in the south most border with Hong Kong. The climate prevalent in our province is more similar to tropics which are in favor of most soil-dwelling parasites. Other factors which explain the high endemicity are increased rate of migration of rural population including the students to crowded cities and cross-border travel and immigration of a considerably large population including many foreigners.
